# Long term follow-up of drug resistant and drug susceptible tuberculosis contacts in a Low incidence setting

**DOI:** 10.1186/1471-2334-12-266

**Published:** 2012-10-22

**Authors:** James Johnston, Andrew Admon, Amir Ibrahim, Kevin Elwood, Patrick Tang, Victoria Cook, Mark Fitzgerald

**Affiliations:** 1 Division of Tuberculosis Control, British Columbia Centre for Disease Control, Vancouver, BC, Canada; 2 Department of Medicine, University of British Columbia, Vancouver, BC, Canada; 3 Department of Internal Medicine, University of Michigan Health System, Ann Arbor, MI, 48109, USA; 4 Department of Pathology & Laboratory Medicine, University of British Columbia, Vancouver, BC, Canada

**Keywords:** Tuberculosis, Multidrug-resistant, Contact investigation, Epidemiology, Latent tuberculosis

## Abstract

**Background:**

Studies examining the transmission of multidrug-resistant tuberculosis (MDR-TB) strains have yielded conflicting results.

**Methods:**

We examined transmission of MDR-TB strains using contact tracing data from a low incidence setting. Contacts of MDR-TB cases diagnosed in British Columbia, Canada, from 1990-2008 were identified through a provincial tuberculosis (TB) registry. Tuberculin skin test (TST) results and TB disease incident rates were determined for contacts. For comparison, TB disease incident rates and TST results were measured in close contacts of isoniazid mono-resistant (HMR-TB) and drug susceptible TB (DS-TB) cases.

**Results:**

Of 89 identified close contacts of MDR-TB patients, 5 patients (6%) developed TB disease and 42 (47%) were TST positive. The incidence rate of TB disease (3%, p = 0.31) and TST positivity (49%, p = 0.82) were similar in contacts of HMR-TB cases. Compared with MDR-TB contacts, DS-TB contacts had lower incidence rate of TB disease (2%, p = 0.04) and TST positivity (32%, p < 0.01). All MDR-TB contacts with culture positive TB diagnosed in follow-up were drug-susceptible; three of six HMR-TB contacts with culture positive TB were HMR-TB. Multivariate analysis demonstrated that contact with MDR-TB (adjusted OR 1.72; 95%CI 1.05-2.81) and HMR-TB (adjusted OR 1.99; 95%CI 1.48-2.67) was associated with TST positivity. In addition, adult age, male gender, BCG positivity, source case sputum smear positivity, foreign birth and fewer contacts per source case were significantly associated with TST positivity in the multivariate model.

**Conclusion:**

Contacts of MDR-TB and HMR-TB patients in a low incidence setting show high rates of TST positivity and TB disease but low rates of drug resistance.

## Background

Multidrug-resistant tuberculosis (MDR-TB) refers to disease caused by *Mycobacterium tuberculosis* strains with *in vitro* resistance to both isoniazid (INH) and rifampin, two first line anti-tuberculosis drugs. The World Health Organization (WHO) estimates that there were 650,000 MDR-TB cases worldwide in 2008, corresponding to 3.6% of all tuberculosis (TB) cases [1]. MDR-TB is associated with high rates of patient default, treatment failure and death [2]. Moreover, MDR-TB treatment is more costly when compared with first line therapy and requires enhanced clinical and laboratory support [1]. For these reasons, MDR-TB is a threat to achieving successful TB control [3].

Transmission of MDR-TB strains has been examined from several perspectives, including *in vitro* and animal studies, mathematical modeling and epidemiological investigation [4]. Results from epidemiological studies have been variable [4,5]. In studies utilizing molecular typing, drug-resistant strains demonstrate both increased and decreased clustering [6-12], while data from traditional contact tracing studies note variable proportions of tuberculin skin test (TST) positivity and TB disease in MDR-TB contacts [13-20].

Contact tracing studies consistently report a significant proportion of contacts with TB disease that demonstrate a distinct resistance profile from their identified MDR-TB source, indicating that not all supposed transmission events involve MDR-TB strains [12,14-20]. Despite this discrepancy, there is considerable evidence to support human-to-human MDR-TB strain transmission. Indeed over half of global MDR-TB cases are thought to result from primary transmission [21]. Yet appropriate preventative treatment of MDR-TB contacts remains unclear, as the relative infectivity MDR-TB strains is unknown and no high quality evidence exists to guide treatment of latent TB infection (LTBI) in contacts of drug resistant cases [22].

We examined local MDR-TB contact tracing outcomes in a low incidence setting. To better understand the transmissibility of MDR-TB strains, the proportion of close contacts of MDR-TB source that developed TB disease or were found to be TST positive in follow-up was compared to the proportion of close contacts of drug-susceptible TB (DS-TB) and isoniazid mono-resistant TB (HMR-TB).

## Methods

### Setting

The study was conducted in British Columbia (BC), a Canadian province with a population of 4.4 million and a TB case rate of 7.1 per 100 000 population per year [23]. The BC Centre for Disease Control (BCCDC) maintains a provincial population based TB registry that is informed of all TB cases through legal notification, as well as case notification through a centralized provincial mycobacteriology laboratory and pharmacy. The same agency dispenses all medications used to treat contacts. Contact tracing was performed according to Canadian guidelines and was recorded in the registry using standardized protocols [24].

### Bacterial culture and susceptibility testing

*M. tuberculosis* was isolated from clinical specimens using the BacT/Alert mycobacterial culture detection system (BioMerieux, Durham, NC). Phenotypic susceptibility testing was performed using the BACTEC 460 radiometric method and interpreted as per Clinical and Laboratory Standards Institute (CLSI) recommendations [25,26].

### Definitions

For the purposes of this study, a *source case* was defined as the first household member to present with culture-positive pulmonary TB and drug susceptibility profile results. The source case date of diagnosis was defined as the date that the first culture positive sample was received by the laboratory. A *close contact* was defined as any individual identified as a “household contact” or “Type 1 contact” of a source case in the provincial TB registry. In this registry, both “household contact” and “Type 1 contact” represent a close contact and refer to household contacts or those sharing airspace with the source case for >4 hours per week. TSTs were performed according to Canadian guidelines [24]. A *positive TST* in a given contact was defined as any TST ≥5 mm measured <3 months before to <1 year after source diagnosis. *Prior positive* referred to a positive TST ≥3 months before source diagnosis. A close contact identified by the source case without a TST measurement or TB disease diagnosis was recorded as *no result*. Contacts with a positive TST or with signs and symptoms of TB disease were screened with a chest x-ray and in some cases, sputum examination. C*ontacts with TB disease* was defined as a contact with *M. tuberculosis* complex demonstrated on culture or an individual with radiological, pathological or therapeutic responses consistent with TB disease as per established guidelines. Contacts with TB disease were not classified by their TST result.

### Data acquisition and analysis

We accessed the BCCDC registry for all cases of MDR-TB, HMR-TB and DS-TB diagnosed between 1990 and 2008. Close contacts of all MDR-TB cases were identified from the registry and their demographic profile, clinical features and TST results were recorded. For comparison, close contacts of DS-TB and HMR-TB were also identified with data recorded in a similar fashion. Only close contacts were recorded to limit bias introduced by enhanced contact tracing of drug-resistant sources. To further limit bias introduced by misclassification of close contacts, any sources with >15 close contacts were excluded from analysis.

The number of contacts with TB disease was recorded, and when available, contact resistance profiles were compared with their purported source case. TST positive or TST negative contacts with two sources listed within the same household in the same year were excluded from further TST analysis. BCG status was extracted for each contact, along with demographic variables. When BCG status was unavailable (in 36% of cases), BCG status was estimated from http://www.bcgatlas.org using data on country of origin, age and year of entry to BC [25,26].

### Statistical analysis

Descriptive statistics were computed using Stata version 11.1 (StataCorp, College Station, Texas). The Wilcoxon Rank Sum test, chi-square test and Ficher’s exact test were used in univariate analysis to assess statistical difference between variables (alpha 0.05). A multivariable logistic regression model was constructed to test the association between source resistance profile and TST positivity. Factors known to predict TST positivity, including adult age (≥18 years), source smear status (positive), male gender, foreign birth, and BCG status were included in the model. After analysis of contact data, the number of contacts per source was added to the model, as the number of contacts varied significantly between HMR contacts and DS-TB contacts. A second model was constructed which excluded BCG status given the limited data for this variable. Odds ratios with 95% confidence intervals were reported. Models were assessed with chi-square goodness-of-fit and ROC curve construction.

## Results

### MDR-TB population

We identified 35 MDR-TB cases from the BCCDC registry between 1990 and 2008 (Table 1). Contact tracing was not performed for 7 cases: 5 had extra-pulmonary TB, 1 case entered BC on MDR-TB therapy, and 1 case left BC before contact tracing was initiated. From the remaining 28 MDR-TB sources, 89 close contacts were identified, with a median of 3 total contacts per source (range 1-7) and median follow-up of 123 months (range 19-239). Of the 89 close contacts, 42 (47%) were TST positive, 33 (37%) were TST negative and 9 (10%) were prior positive or no result (Table 2). Latent TB therapy was completed in 12 MDR-TB contacts, including 11 contacts that initiated preventative therapy tailored to the source case susceptibility profile. Five close contacts developed TB disease during follow-up; fully susceptible *M. tuberculosis* was isolated from 4 cases and the fifth case was diagnosed based on clinical criteria. All 5 contacts with TB disease were diagnosed within 3 months of source diagnosis and had not received preventative therapy.

**Table 1:** Characteristics of TST positive and negative contacts

	**DS-TB (%)**	**HMR-TB (%)**	**MDR-TB (%)**	**p-value vs DS-TB**	
				**HMR-TB**	**MDR-TB**
Source Cases	2895	96	28		
Household contacts	7309	249	89		
Median contacts/source	3	3	3	<0.001^	0.839^
**Source Characteristic**					
Source Smear Positive	4709 (64)	139 (56)	57 (64)	0.005	0.939
**Contact Characteristics**					
**Demographics**					
Mean Age (Sd)	32.9 (21.2)	28.9 (20.4)	26.7 (19.1)		
Age Over 18	5489 (75)	170 (68)	57 (64)	0.013	0.015
Male Gender	3521 (48)	112 (45)	43 (48)	0.32	0.98
**Birth Country**					
Canadian born	3163 (43)	87 (35)	60 (67)		
Foreign born	3609 (49)	154 (62)	23 (26)		
Unknown	536 (7)	8 (3)	6 (7)		
BCG status*					
Positive	3340 (46)	132 (53)	44 (49)		
Negative	2977 (41)	94 (38)	42 (47)		
Unknown	991 (14)	23 (9)	3 (3)		

^Wilcoxon rank sum test.

* 22% of values were calculated using age, country of origin and information from http://www.bcgatlas.org.

**Table 2:** Results of contact tracing

	**DS-TB (%)**	**HMR-TB (%)**	**MDR-TB (%)**	**p-value vs DS-TB**	
				**HMR-TB**	**MDR-TB**
Source cases	2895	96	28		
Close contacts	7309	249	89		
TST positive	2321 (32)	121 (49)	42 (47)	<0.001	0.002
Secondary case	168 (2)	8 (3)	5 (6)	0.347	0.039
Prior positive	448 (6)	12 (5)	8 (9)	0.395	0.265
No result	475 (6)	9 (4)	1 (1)	0.067	0.045

### Isoniazid-resistant and drug-susceptible populations

Between 1990 and 2008, contact tracing of 96 infectious HMR-TB source cases yielded 249 close contacts, of whom 121 (49%) were TST positive (Table 2) and 8 (3%) developed TB disease during follow-up. Of the six contacts with culture-confirmed TB, 3 developed HMR-TB, while 3 developed DS-TB (Table 3). DS-TB contact tracing over the same period yielded 7309 close contacts from 2895 sources (Table 2). There were 2321 contacts (32%) with a positive TST and 168 (2%) contacts with TB disease. Despite a common median of 3 close contacts per source case, the distribution of contacts varied significantly between DS-TB and HMR-TB (p < 0.001).

**Table 3:** Drug resistance patterns in source and secondary cases

**Related pairs**	**Resistance pattern**			
	**Isoniazid**	**Rifampin**	**Ethambutol**	**Streptomycin**
MDR-TB1	0.1	2.0	4.0	S
	S	S	S	S
MDR-TB2	0.1	2.0	NR	S
	S	S	S	S
MDR-TB3	0.1	2.0	4.0	S
	S	S	S	S
MDR-TB4	0.4	2.0	2.5	S
	S	S	S	S
MDR-TB5	0.4	2.0	2.5	S
	S	S	S	S
HMR-TB1	0.1	S	S	S
	0.1	S	S	S
HMR-TB2	0.1	S	S	S
	0.1	S	S	S
HMR-TB3	0.1	S	S	S
	S	S	S	S
HMR-TB4	0.1	S	S	S
	S	S	S	S
HMR-TB5	0.1	S	S	S
	0.1	S	S	S
HMR-TB6	0.1	S	S	S
	S	S	S	S

The first row in each pair represents source case sensitivity profile, while the second represents the secondary case susceptibility. *S* = sensitive, *NR* = no result. Resistance values reported in microG/microL.

### Multivariate analysis of TST results

All seven pre-specified variables predicted TST positivity in the logistic regression model, including adult age (OR 1.76; 95% CI 1.51-2.06), male gender (OR 1.18; 95% CI 1.05-1.32), BCG vaccination (OR 1.42; 95% CI 1.23-1.64), foreign birth (OR 5.37; 95% CI 4.55-6.33), source smear positivity (OR 1.23; 95% 1.09-1.39), source HMR-TB (OR 2.13; 95% CI 1.57-2.90), and source MDR-TB (OR 1.75; 95% CI 1.07-2.86) (Table 4). The number of close contacts attributed to the index case was included in the model because the distribution varied significantly between the HMR-TB and DS-TB groups, and because a lower threshold for classifying contacts as Type 1 may impact the rate of TST positivity. Each single increase in the number of close contacts was associated with a decrease in TST positivity (OR 0.96; 95% CI 0.94-0.98). Estimates did not change significantly after BCG status was excluded from the model. Chi-square goodness of fit was non-significant for models that included and excluded BCG status, indicating that the goodness-of-fit for this model appeared adequate (p = 0.32, p = 0.09 respectively).

**Table 4:** Multivariate analysis of factors associated with TST positivity

**Variable**	**Adjusted OR**	**p-value**	**95% CI**
Age over 18	1.77	<0.001	1.53 - 2.06
Male gender	1.19	0.002	1.07 - 1.33
Foreign birth	6.97	<0.001	6.11 - 7.95
HMR-source	1.99	<0.001	1.48 - 2.67
MDR-source	1.72	0.030	1.05 - 2.81
Smear positive source	1.23	0.001	1.09 - 1.38
Increase in source contacts (per contact)	0.96	<0.001	0.95 - 0.98

## Discussion

Guidelines addressing latent TB infection (LTBI) treatment in MDR-TB contacts are vague and somewhat contradictory [24,27,28]. The WHO, citing the unknown efficacy of tailored preventative regimens, recommends observing contacts with careful clinical follow-up over two years [28]. The American Thoracic Society recommends observation or treatment with one of two regimens for 6-12 months [27]. These recommendations reflect the lack of high quality evidence required to direct decisions. Indeed, a recent Cochrane review failed to identify randomized control trials on MDR-TB contact tracing, while a 2006 systematic review found only two comparative studies suitable for analysis [23,29].

Given our results, combined with the discouraging completion rates of susceptibility-profile tailored regimens [30-32], IPT could be seen as a potential option in close contacts of MDR-TB patients in low incidence settings. This strategy, however, is not without risk [33]. MDR-TB transmission does occur in low incidence settings [12]. Moreover, in high incidence settings, the majority of patients with TB disease in MDR-TB contact populations develop MDR-TB [16,18,19]. Such contacts will not likely benefit from IPT. More importantly, however, this strategy could propagate drug-resistant disease by selectively killing drug sensitive organisms [34-37]. Thus, the risk of IPT appears to outweigh its benefit in MDR-TB contacts.

Our results demonstrate that MDR-TB contacts have higher rates of TST positivity compared with DS-TB contacts. This may reflect the increased transmissibility of MDR-TB strains. Alternatively, the high rates of TST positivity in MDR-TB and HMR-TB contacts may reflect differential distribution of unmeasured determinants for LTBI, such as source time-to-diagnosis, source cavitary disease or contact environmental and socioeconomic determinants. Indeed the development of DS-TB in all close contacts of MDR-TB with incident TB disease supports the notion that close contacts of drug-resistant source cases may be at higher risk for LTBI and TB disease independent of transmission from an identified MDR-TB source case.

There are several limitations in our study, the most significant being the lack of molecular typing data, which could help determine whether the discrepant drug susceptibility profiles were from strains with the same genotype. A second limitation is the small population of drug-resistant cases and contacts available for analysis. Previous studies examining traditional contact tracing in MDR-TB patients have demonstrated disease rates ranging from 0-8% in close contacts, consistent with our data [10-19]. However, in studies examining drug-susceptibility profiles, 62-92% of contacts that subsequently developed active disease had MDR-TB [13-16,18-20]. These rates are consistent with data demonstrating that 70% of secondary cases have the same genotype as their source case [38].

Finally, data on several determinants for TB infection are absent from analysis, including time-to-diagnosis, socioeconomic status and medical co-morbidities. Differential rates of TST positivity may be, in part, related to the differential distribution of these determinants. Nonetheless, our data suggests that risk for *M. tuberculosis* infection is higher in the contacts of drug resistant cases, and that thorough contact tracing should be performed in this population.

## Conclusions

In conclusion, we described our experience with the contact tracing results of drug-resistant TB in a low prevalence region over nearly two decades. Our findings demonstrate that close contacts of MDR-TB at higher risk for LTBI and active TB. We suggest enhanced contact tracing in MDR-TB contacts, but caution against IPT in this population. Further research is urgently needed to determine the optimal management of LTBI in MDR-TB contacts.

## Abbreviations

TB: Tuberculosis disease (also known as active tuberculosis); LTBI: Latent tuberculosis infection; MDR-TB: Multidrug resistant tuberculosis; DS-TB: Drug sensitive tuberculosis; HMR-TB: Isoniazid mono-resistant tuberculosis; INH: Isoniazid; IPT: Isoniazid preventative therapy; WHO: World Health Organization; TST: Tuberculin Skin Test.

## Competing interests

The authors declare that they have no competing interests.

## Authors’ contributions

JJ: design, data acquisition, writing, statistical analysis. AA: statistical analysis, writing. AI: data acquisition. writing. PT: data acquisition, writing. VC: design, writing. KE: design, writing. MF: design, writing. All authors read and approved the final manuscript.

## Pre-publication history

The pre-publication history for this paper can be accessed here:

http://www.biomedcentral.com/1471-2334/12/266/prepub
